# IPM Strategy to Control EFB in *Apis mellifera*: Oxytetracycline Treatment Combined with Partial Shook Swarm and Queen Caging

**DOI:** 10.3390/vetsci11010028

**Published:** 2024-01-10

**Authors:** Michela Mosca, Andrea Gyorffy, Marco Pietropaoli, Luigi Giannetti, Antonella Cersini, Luca Fortugno, Giovanni Formato

**Affiliations:** 1 Istituto Zooprofilattico Sperimentale del Lazio e della Toscana “M. Aleandri”, Via Appia Nuova 1411, 00178 Rome, Italy; michela.mosca-esterno@izslt.it (M.M.); andrea.gyorffy-esterno@izslt.it (A.G.); marco.pietropaoli@izslt.it (M.P.); luigi.giannetti@izslt.it (L.G.); antonella.cersini@izslt.it (A.C.); 2 Servizi Veterinari ASL di Viterbo, Via Vincenzo Cardarelli SNC, 01100 Viterbo, Italy; luca.fortugno@asl.vt.it

**Keywords:** European foulbrood, oxytetracycline, partial shook swarm, queen caging, residues, relapses, integrated pest management

## Abstract

**Simple Summary:**

To control the most devastating honey bee diseases while decreasing the application of chemicals, integrated pest management strategies can be applied. These strategies favor the combination of chemical and non-chemical approaches. European foulbrood is one of the most prominent infectious honey bee diseases that has been treated with antibiotics in the past decades. To test the efficacy of the combination of chemical (antibiotics) and non-chemical (beekeeping management techniques) treatments, we treated the honey bee colonies suffering from European foulbrood with a combination of antibiotics, namely oxytetracycline and management techniques, i.e., partial shook swarm and queen caging. To assess the potential added value of the combination of treatments to the purely non-chemical intervention, in the control group, we only applied partial shook swarm and queen caging but no oxytetracycline. We have been monitoring the feeding and strength of the colonies, the side effects of the treatments, the relapses of the disease, and the amount of antibiotic residues in the honey yield for 7 months. Treating the honey bees with oxytetracycline resulted in weaker colonies, subclinical—therefore easy to carry over—disease cases, and higher levels of antibiotic residues in the honey yield. The applied management techniques led to adverse side effects with or without combining them with the antibiotic. Two months after the administration of the antibiotic, the level of the residues was lower than the maximum residue limit allowed by EU legislation.

**Abstract:**

We tested an integrated pest management (IPM) strategy to control European foulbrood (EFB) in honey bees. Colonies affected by EFB were assigned to two homogenous groups: an oxytetracycline-treated group (1.5 g OTC/hive) that underwent partial shook swarm (PSS) in combination with queen caging (QC) and an untreated group where only two beekeeping techniques, PSS and QC, were applied. The consumption of sucrose solution, the strength of the colonies, side effects of the mentioned techniques, clinical as well as subclinical relapses of EFB, and the amount of OTC residues in the honey were assessed over a 7-month-long monitoring period. Regarding the consumption of the sucrose solution, there was no significant difference between the OTC-treated and untreated groups. The strength of the untreated colonies was consistently but not significantly higher than those treated with OTC. PSS combined with QC resulted in various side effects in both groups: queen loss (52%), absconding (8%), and drone-laying queen (4%). Untreated colonies (16.7%) showed clinical EFB relapses 4 months after the application of PSS along with QC, while 15.4% of the OTC-treated colonies were confirmed EFB-positive by PCR. OTC residues were detected in the honey yielded in the cases of both groups. Two months after the PSS, the amount of OTC residues in the untreated group was 0.6 ± 0.2 µg/kg, while that in the OTC-treated group amounted to 5.8 ± 11.6 µg/kg; both results are below the maximum residue limit (MRL) of 100 ppb considered in the EU for cascade use.

## 1. Introduction

European foulbrood (EFB) is a worldwide bacterial disease of *Apis mellifera* larvae caused by a Gram-positive bacterium, *Melissococcus plutonius* (*M. plutonius*). EFB is often associated with comorbidities caused by *Paenibacillus alvei*, *Brevibacillus laterosporus*, and *Enterococcus faecalis*. EFB causes severe economic losses in the apiculture sector, affecting not only the health of honey bees but also the international trade of beehive products [1,2].

The prevalence and clinical manifestation of EFB varies across geographical conditions and is mostly impacted by the climate and the quality of feed [3,4]. Some *M. plutonius* strains show high virulence associated with a plasmid carrying the gene encoding melissotoxin A. The presence of such a gene in the *M. plutonius* isolate predicts honey bee brood mortality [5]. In Europe, EFB is widespread in Great Britain [6], while it is endemic in Switzerland, showing an increasing incidence since the late 1990s [7]. EFB outbreaks have been recently reported in the Czech Republic, Finland, France, Greece, Norway, the Netherlands, Switzerland, and the United Kingdom [8]. Seventy-nine countries worldwide classified EFB as a notifiable disease [5]. The presence of *M. plutonius* has been confirmed in five continents. Among the member states of the World Organization for Animal Health (WOAH), Nicaragua and Mozambique are the only nations to have never reported the presence of EFB by 2019 [9].

EFB control is based on applying broad-spectrum antibiotics that are authorized for treating honey bees [10,11,12]. Among those, oxytetracycline (OTC)-containing products are the most widespread in beekeeping for controlling various bacterial honey bee diseases, resulting in increasing rates of antibiotic resistance (ABR) [5,10,13]. OTC-resistant strains of *M. plutonius* have already been isolated in Canada [11]. In China, such resistant strains are reported to be ubiquitous [14]. To prevent the development of ABR and the occurrence of antibiotic residues in honey, the application of antibiotics to honey bees has been banned in many countries [15], including the EU Member States, where honey bees can only be treated with OTC in an off-label manner, via the cascade principle [16,17]. If OTC is applied via the cascade principle, the maximum residue limit (MRL) for OTC residues in the honey is 100 µg/kg [18,19]. According to reports from the European Food Safety Authority (EFSA), 1–7% of honey samples of EU origin contained antibiotic residues, which might be attributed to the reluctance of EU beekeepers to avoid using antibiotics for treating honey bees [20]. Shendy et al. (2016) reported that before 2016, ∼71% of the EU Rapid Alert System for Food and Feed (RASFF) notifications listed the presence of antibiotic residues in honey bee products originating from Belgium, China, Poland, and Romania, where one-fourth of those notifications concerned nitrofurans and nitroimidazoles [21]. Such residues can occur not only in the honey but also in other hive products [15,22,23]. According to the RASFF window [24], during 2022 (“last year”), there were two notifications for antibiotics in the product category “honey and royal jelly” in the case of EU countries as the countries of origin. The objects of the above notifications were “Chloramphenicol in honey from Poland (origin Romania)” and “Dihydrostreptomycin in acacia honey”, respectively.

In humans, the residues of OTC, acting at the sub-therapeutic level, might lead to acute or chronic conditions, e.g., allergic reactions, liver damage, dysbiosis, gastrointestinal disorder [15], and adverse reactions in the bones and teeth in children [25]. Antibiotic residues occurring in the food and feed can lead to the advancement of ABR [26] by exerting selection pressure on the natural adaptation feature of the bacteria. Antibiotic residues can pollute the environment via water and soil as well as by entering the food chain, while they can also be deposited in the dust, hence also spreading by transportation. Genes coding ABR against OTC can also spread horizontally via mobile genetic elements (e.g., plasmids or transposons). On a host population level, such bacteria can be transmitted by social behavior and the environment [12].

The most widespread methods to control EFB are the destruction of the whole colony, shook swarm techniques, antibiotic treatment, and specific interventions based on integrated pest management (IPM) strategies, as defined by FAO: “IPM is the careful consideration of all available pest control techniques and subsequent integration of appropriate measures that discourage the development of pest populations. It combines biological-, chemical-, physical-, and crop-specific (cultural) management strategies and practices to grow healthy crops and minimize the use of pesticides, reducing or minimizing risks posed by pesticides to human health and the environment for sustainable pest management” [27]. Among such IPM strategies applied for the beekeeping sector, it is believed that antibiotic treatments should be combined with good beekeeping practices, e.g., with the shook swarm method.

The shook swarm method can be either complete or partial, based on the management of the combs. In the case of a “total” shook swarm (TSS), all the combs are changed to new ones. Therefore, TSS is an efficient method to reduce the level of EFB infection; however, it can cause serious side effects (e.g., the formation of orphan colonies); thus, beekeepers tend to avoid applying TSS so as to prevent losing the honey harvest season. During a “partial” shook swarm (PSS), only the brood combs are removed and replaced by new ones, while the storage combs are left intact. In the case of EFB, beekeepers prefer to undertake PSS, removing only the affected combs of brood, thus promoting the recovery of the colony. To reduce the risk of losing the queen (about 10%, [28]), queen caging (QC) was implemented before the shaking of the frames could be applied while performing PSS.

We present the results of this study, aiming at evaluating the efficacy of PSS combined with QC, per se or complemented by the administration of OTC for controlling EFB in Central Italy. Consumption of the medicated or intact sucrose solution, as well as the colony strength, side effects, EFB relapses, and OTC residues in honey, were assessed over the 7-month-long monitoring period.

## 2. Materials and Methods

### 2.1. Experimental Setup and Preparation of the Sucrose Solution

The field trial was conducted in collaboration with the local public veterinary services in an experimental apiary of 25 colonies naturally affected by EFB between April and December 2022. To confirm EFB, an on-field EFB Diagnostic Kit (Vita Europe Ltd., Basingstoke, UK) and bacterial isolation and identification, according to the official WOAH detection guidelines [29], were applied.

To assign the colonies into two homogenous experimental groups, one OTC-treated group, and one untreated, colony strength and the infection rate were assessed. Colony strength was determined based on counting (1) the number of frames covered by adult honey bees and (2) the number of frames occupied by brood. The infection rate was judged by estimating the number of affected larvae on each side of every comb. The number of affected larvae on each comb was estimated using a square-shaped cardboard equivalent to the size of 10 × 10 cells, placed on an area of uncovered brood. On a 0 to 3 scale, the number of affected larvae was given as terciles (0, 1–33, 34–65, 66–100) [30].

Each of the 13 beehives belonging to the OTC-treated group was administered 3.2 mL of the pharmaceutical product Terramicina 100 (Zoetis Italia S.r.l., Rome, Italy), which contains OTC hydrochloride, mixed in 180 mL of 1:1 sucrose solution semel in die, for five consecutive days [31]. Thus, each colony received 1.5 g OTC in total over the treatment period. The 12 colonies of the untreated group received the same amount of pure sucrose solution over the same time period. PSS was applied to both groups two days after the last OTC treatment. The infected brood combs were destroyed. During the PSS, queens were caged together with 5 nurse honey bees to reduce the risk of unintentional damage/killing or the escape of the queen. The queen cage, plugged with candy, was placed into the beehive after the PSS had been performed. The queen was released from the cage as the adult honey bees consumed the candy cork, occurring within a few days after having placed the queen cage into the beehive.

During the field trial, the following parameters were investigated: consumption of the sucrose solution (mL/day), the strength of the honey bee colony (number of frames covered by adult honey bees as well as brood), side effects linked with the PSS + QC technique (presence of the queen, absconding, mother drone colonies), clinical and subclinical relapses of EFB, the amount of honey produced (kg/colony), and the amount of OTC residues in the honey (µg/kg).

### 2.2. Assessing Consumption of Sucrose Solution

The consumption of the sucrose solution was measured with a graduated cylinder 24 h after each administration.

### 2.3. Assessing Colony Strength

Colony strength was evaluated according to the number of combs covered by honey bees as well as by brood in each beehive at three time points during the monitoring period: one week, one month, and two months after the application of PSS.

### 2.4. Inspection of the Beehives

The experimental apiary was investigated with the support of the local official veterinarians over the 7-month monitoring period every 2 weeks. The possible side effects of the IPM (queen loss, absconding, drone mother colony), clinical signs of EFB relapses, and the amount of honey yield in the honey chambers were recorded one week, and 1, 2, 4, and 7 months after PSS.

### 2.5. Detecting Clinical and Subclinical Relapses of EFB

To detect possible subclinical relapses, real-time PCR screening for a gene sequence specific to *M. plutonius* was applied. From each beehive, samples of 20 adult honey bees were taken. The honey bees were homogenated in 20 mL of 1× PBS, and the nucleic acids were extracted by the MagPurix Viral/Pathogen Nucleic Acid Extraction Kit B (Cat. ZP02012, ZINEXTS, New Taipei City, Taiwan) in an automated extractor. The real-time PCR protocol specific for *M*. *plutonius* [7] targeted a 79 bp region in the sodA gene. The master mix was performed with the TaqMan^®^ Universal PCR Master Mix kit (Applied Biosystems, Life Technologies, Waltham, MA, USA; Cat. No. 4318157). The master mix consisted of 12.5 µL of TaqMan Universal PCR Master mix 2× buffer and 0.3 µM (0.25 µL) of the forward primer (Melisso fw: 5′-CAGCTAGTCGGTTTGGTTCC-3′) and the reverse primer (Melisso Rv: 5′-TTGGCTGTAGATAGAATTGACAAT-3′), both at an initial concentration of 30 µM. Further, 0.1 µM (0.25 µL) of the probe at the starting concentration of 10 µM, 5 µL of mold (DNA), and 6.75 µL of sterile H_2_0 GR was used for a total reaction volume of 25 µL. Real-time PCR was performed using QuantStudio 7 Flex (Applied Biosystems, Foster City, CA, USA) while applying the following thermal cycle: 50 °C for 2 min, 95 °C for 10 min, 95 °C for 15 s in 40 cycles, and 60 °C for 1 min.

### 2.6. Measuring the Residues of OTC in the Honey

For assessing the amount of OTC residues in the honey, a piece of uncapped nest comb (5 cm × 4 cm) was removed from under the spot where the Petri dishes containing the medicated (OTC-treated group) or untreated (untreated group) sucrose solution were placed. Samples were taken before the OTC treatment, as well as one week, one month, and two months after the PSS, respectively. Samples were investigated according to the method described by Giannetti et al. [32] with slight modifications. An aliquot of 2 g of the honey sample was weighed in a 15 mL centrifuge tube and mixed with 10 mL McIlvaine-EDTA for 15 min in an ultrasonic bath. The extract, after centrifugation, was charged on an Oasis HLB (60 mg) SPE cartridge that was previously activated with 3.0 mL of methanol and 3.0 mL of deionized water. The SPE cartridge was washed with 3.0 mL of deionized water and 2.0 mL of methanol-water 5:95 *v*/*v*. Tetracyclines were eluted with 3 mL of methanol containing 0.1% formic acid, and the eluted liquid was evaporated at 40 °C under a nitrogen stream. The residue was finally dissolved in 0.5 mL of methanol-water 50:50 *v*/*v* and injected into the LC-MS/MS system. Analyses were carried out with a QTRAP 5500 tandem mass spectrometer detector (AB Sciex, Redwood City, MA, USA) equipped with a 1260 Infinity high-performance liquid chromatograph (Agilent Technologies, Santa Clara, CA, USA). The instrument was set to a positive electrospray ionization mode with a capillary voltage of 5.5 kV and a source temperature of 550 °C. Ultra-pure air was used as the nebulizer gas, and ultra-pure nitrogen was applied both as a curtain and collision gas. Positive ions were acquired in the multiple-reaction monitoring (MRM) mode, acquiring two or more diagnostic product ions from the chosen precursors to obtain high specificity and sensitivity. The chromatographic separation of the analytes was achieved on a Luna C18 column (100 mm × 2.0 mm, 2.5 µm, Pnenomenex, Santa Clara, CA, USA) with a mobile phase consisting of 0.1% formic acid in water (mobile phase A) and acetonitrile (mobile phase B) at a flow rate of 0.3 mL min^−1^ in the gradient mode. The limit of detection (LOD) of the method was 0.1 µg/kg for the tetracyclines and their epimers.

Colony strength data, as well as the amount of OTC residues in honey, were analyzed using the XLSTAT™ 2023.1.6 1410 software [33], applying Wilcoxon’s signed-rank test with a two-tailed distribution, respectively. The effect of the localization of the hives was analyzed by applying simple repeated measures ANOVA.

## 3. Results

### 3.1. Consumption of the Sucrose Solution

All honey bee colonies in both the OTC-treated and untreated groups consumed all of the 180 mL sucrose solution after each administration.

### 3.2. Colony Strength

The strength of the untreated colonies was constantly higher than that of the OTC-treated ones, albeit the difference was never significant (Table 1 and Table 2).

### 3.3. Side Effects of the IPM: Partial Shook Swarm + Queen Caging

During the 7-month monitoring period of the beehives, we observed the following side effects after applying PSS combined with QC: queenless colonies, absconding colonies, and drone mother colonies. A total of 16 out of 25 honey bee colonies (both OTC-treated and untreated) had to be considered dropouts during the monitoring period due to the side effects of PSS combined with QC.

Two (15.4%) out of thirteen colonies absconded as a result of non-reproductive swarming in the OTC-treated group during the first week after PSS. In 1 out of 12 colonies (8.3%) of the untreated group, a drone mother colony had developed by the second month of sampling following the PSS. Queen loss was observed in 7 out of 12 (58%) colonies of the untreated group and 6 out of 13 (46.2%) colonies in the OTC-treated group. Five queens out of twenty-five disappeared immediately after performing the PSS, with five more during the first month and three more during the second month after the PSS (Table 3).

### 3.4. Clinical and Subclinical EFB Relapses

#### 3.4.1. Clinical EFB Relapses

Clinical relapses were screened during the 7-month monitoring period. Four months after performing the PSS, 2 out of 12 (16.7%) colonies showed EFB clinical symptoms in the untreated group, while no clinical relapses were detected in the OTC-treated group (Table 4).

#### 3.4.2. Subclinical EFB Relapses

The subclinical presence of *M. plutonius* was investigated by PCR in nurse honey bee samples seven months after the PSS. In the OTC-treated group, in 2 out of 13 colonies (15.4%), the presence of *M. plutonius* DNA was confirmed. None of the 12 untreated colonies tested positive for *M. plutonius* by PCR 7 months after the PSS.

### 3.5. Honey Yield

No honey was stored in the honey chambers in either colony of either group during the monitoring period.

### 3.6. Amount of OTC Residues in the Honey Samples

All honey samples taken before OTC treatment in both experimental groups proved to be negative for OTC residues.

**Figure 1:**
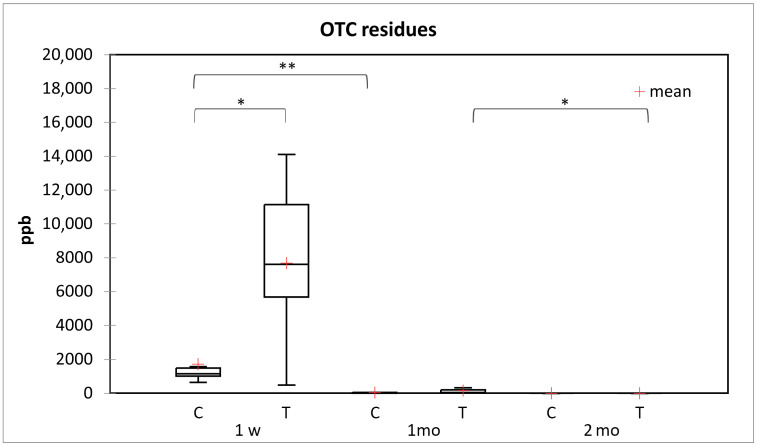
Average amount of OTC residues (µg/kg) in honey samples taken from the OTC-treated (T) and the control groups (C). The samples were collected 1 week (1w), 1 month (1mo), and 2 months (2mo) after the PSS, respectively. The level of significance is indicated by asterisks (* *p* ≤ 0.05, ** *p* ≤ 0.01).

One week after the PSS, the average amount of OTC residues in the honey samples taken from the OTC-treated colonies was 7679 ± 4579 µg/kg, while in the untreated group, it amounted to 1719 ± 1605 µg/kg (Figure 1). One month after the PSS, the average amount of OTC residues in the honey samples was 156.9 ± 217.5 µg/kg in the OTC-treated group and 46.2 ± 55.3 µg/kg in the untreated group, respectively. By the end of the second month during the monitoring period, the average amount of OTC residues detected in the honey samples collected from the OTC-treated group was 5.8 ± 11.6 µg/kg, while in the samples collected from the untreated colonies, it amounted to 0.6 ± 0.2 µg/kg.

The difference in the amount of the OTC residues between the samples collected from the OTC-treated group and the untreated one was significant (*p* < 0.05) upon the first sampling, i.e., one week after the PSS.

The decrease in the amount of OTC residues in the untreated group was significant between the first week and the first month after PSS (*p* < 0.008). In the case of the OTC-treated group, such a decrease was significant only between the first and the second month (*p* < 0.031) (Figure 1) during the monitoring period.

The queenless colonies of both the OTC-treated and the untreated groups showed a clear yet insignificant trend in comparison with the queen-right colonies. One week after the PSS, the honey collected from the OTC-treated queenless colonies showed a 6 times lower average OTC residue level (1488.0 ± 1431.9 µg/kg) than that originating from the OTC-treated colonies with the right queen (9.743.0 ± 2915.3 µg/kg). Within the untreated group, the average amount of the OTC residues in the honey amounted to 1123.0 ± 457.1 µg/kg in the queenless colonies, while it was 1870.0 ± 1766.9 µg/kg in the queen-right ones.

## 4. Discussion

We tested real-life scenarios on the field to assess the efficacy and benefits of an integrated treatment for controlling EFB and to gauge whether treatment with OTC has an additional value over beekeeping management techniques. While both TSS and PSS can reduce the spillover of OTC in the beehive, PSS was chosen instead of TSS to facilitate colony recovery. QC was applied to prevent queen loss and absconding [34]. The shook swarm method, both TSS and PSS, is reported as an alternative to OTC treatment in the case of EFB. The combination of TSS and OTC treatment was found to reduce the recurrence of EFB at the colony level [9]. We monitored the experimental apiary for 7 months after the PSS, assessing the sucrose solution consumption, colony strength, side effects, honey yield, and relapses, and collected samples for the laboratory investigation (residues of OTC in the honey and real-time PCR analyses for the *M. plutonius* gene sequence). 

### 4.1. Consumption of the Administered Sucrose Solution

Antibiotics can be administered in various ways, such as by mixing them into a sucrose solution or icing sugar and spraying the mixture over empty combs or onto adult honey bees [35]. By applying antibiotics using conventional methods, antibiotic contamination of the beehives, including the wood of combs, the wall of the beehive, and the stored honey, cannot be avoided.

We administered OTC to the honey bees per os, using Petri dishes [31]. In this way, (1) we could reduce the risk of antibiotic contamination within the beehive, including that of the honey yield, and (2) we could quantify the amount of antibiotics consumed by the honey bees.

In agreement with our previous study [28], all the colonies affected by EFB, having more than 3.5 frames covered by adult honey bees, could easily take up all the sucrose solution, with or without OTC. Thus, all the OTC-treated colonies consumed the therapeutic dose of OTC (1.5 g over 5 days) needed to control EFB.

### 4.2. Colony Strength 

The PSS had a highly negative impact on the strength of the colonies, both in the OTC-treated and the untreated groups. However, in comparison with the OTC-treated colonies, untreated colonies remained stronger all along the monitoring period, in line with the results reported in other studies [31,36,37,38].

Both the untreated and the OTC-treated groups showed a significant recovery in the course of the first month after the PSS and a non-significant one during the second month of the monitoring activity. In both groups, colonies were too weak to store surplus honey, even if PSS rather than TSS was applied.

### 4.3. Side Effects of the IPM: Partial Shook Swarm + Queen Caging 

The most often side effects of PSS and/or QC observed were absconding, queen loss, and the development of a mother drone colony. Absconding is a non-reproductive swarming, an evolutionary behavioral response to internal or external stress factors [34]. Queen loss is considered to happen when the absence of the queen is longer than the time period needed for reproduction (3–45 min). In the latter case, the worker honey bees sense the loss of the queen through a pheromone effect and start rearing a new queen from the larvae [39]. In our previous studies, when we performed PSS on 30 colonies in the summer, we had a 10% ratio of queen loss following PSS [28]. We applied QC to further reduce queens’ loss that might occur after the PSS, yet 13 out of 25 colonies lost their queen. The lack of reacceptance and killing of the mother was the cause of queen loss in five colonies in the first month after the PSS and in three colonies during the second month after the PSS. Moreover, 2 out of 13 colonies swarmed during the first week after the PSS in the OTC-treated group, and one drone mother colony developed out of 12 untreated colonies during the second month after the PSS. 

Caging the queen is a highly traumatic event for the colony if performed in spring, especially in combination with the shook swarming technique, even in the case of applying PSS. In spring, the life cycle of the honey bee colony reaches the summit of reproductive activities (natural swarming), representing a physiologically sensitive period. The colonies investigated might have reacted excessively to the segregation of the queen experiencing the physiologically sensitive period. The queen mortality rate due to QC is considered to be acceptable up to 10–12% [40].

### 4.4. Relapses of EFB

Hornitzky et al. [41] reported that an infected colony can reach a balance in regards to EFB infestation due to the cleaning behavior of the nurse honey bees. In this way, EFB infection might persist in the colony with no or scarce symptoms for years. EFB-infected colonies can also recover spontaneously due to the high variability in the virulence of *M. plutonius* [42]. We detected symptomatic EFB 4 months after the PSS in 2 out of 12 beehives in the untreated group and asymptomatic EFB in the OTC-treated group 7 months after the PSS in 2 out of 13 colonies. When the symptoms of EFB appear, detection and intervention are possible immediately in the apiary. The subclinical form of EFB can only be detected by laboratory methods. The colonies in which EFB is persisting are sources of continuous reinfection in the affected apiary and might adversely impact the health status of the neighboring apiaries both directly (through drifting and robbing) and indirectly (moving the beehives, exchange of beekeeping tools between the apiaries). Based on our findings, even treatment with a total amount of 1.5 g OTC applied over 5 days can fail to exert its full therapeutic effect and can lead to asymptomatic EFB. The detection of the *M. plutonius*-specific gene sequence by real-time PCR in the OTC-treated group in month 7 could be the result of the reinfection originating from the untreated colonies that were symptomatic 4 months after the PSS. 

### 4.5. Honey Yield

No surplus honey was stored by any of the colonies involved in the field test.

It should be considered that the colonies were not fed after the PSS and that the weather conditions during the field trial were not favorable for the honey bees: spring frosts/low temperatures and wind followed by summer drought drastically reduced the number of available blooms, negatively interfering with honey production.

The PSS was applied to improve the colony’s strength by leaving storage available, resulting in a faster recovery and an appropriate honey yield during the summer. During our on-field experiment, colony strength was not exclusively affected by EFB and the PSS applied. Due to the unfavorable weather conditions, the blooming might have been restricted, preventing honey bees from collecting appropriate amounts of nectar and pollen. 

### 4.6. Residues of OTC in the Produced Honey

Before the OTC treatment, no OTC residues were detected in the honey collected from the beehives. Already one week after the OTC administration, honey samples collected from both the OTC-treated (7679 ± 4579 µg/kg) and the untreated group (1719 ± 1605 µg/kg) contained OTC residues. The results confirmed the cross-contamination between the OTC-treated and untreated colonies within the experimental apiary. Such cross-contamination with OTC was also reported by other studies [31,43]. In the present trial, the PSS applied 2 days after the end of OTC treatment might have caused a drift between the colonies, resulting in cross-contamination among the beehives.

We detected a quick decrease in the amount of OTC residues that could be detected in the honey samples as compared to other studies where OTC administration was followed neither by TSS nor PSS [31,44]. By the end of the second month during the monitoring period, the amount of OTC residues dropped below the MRL (100 µg/kg) set by the EU legislation in both the OTC-treated (5.8 ± 11.6 µg/kg) and the untreated groups (0.6 ± 0.2 µg/kg). Since we did not feed any of the colonies after the OTC treatment, the honey bees of both groups might have consumed the honey storage contaminated with OTC to build new nest combs after the PSS.

In regards to the impact of the presence of the queen on the amount of OTC residues in the honey produced, we found that the amount of such residues was lower in queenless colonies than in the queen-right ones, regardless of whether they were OTC-treated or untreated. The reason for the latter phenomenon could be that (1) queenless colonies consumed more honey stored and/or might have been more exposed to robbing by the queen-right colonies and/or (2) queen-right colonies are able to collect more nectar, resulting in the dilution of the honey and consequently reducing the OTC residue concentration. 

The cross-contamination between the OTC-treated and untreated colonies could occur through both the drift behavior and the mixing of honey bees belonging to different colonies of the different groups after the PSS. In the case where PSS is performed after the OTC treatment, separating healthy colonies from the symptomatic ones before performing either TSS or PSS is advisable.

Considering the results of the present study, we can provide the following recommendations to minimize the presence of OTC residues in honey and promote the responsible use of antibiotics in beekeeping as well as to facilitate compliance with the relevant legislation:

– The antibiotic should be administered in a medicated solution, preferably in jar cups of Petri dishes enriched with a piece of net to avoid the contamination of the beehive as well as the drowning of the honey bees. The practice also enhances the exact administration of the active ingredient, as well as the control of the consumption and the separation of the excess infected sucrose solution.
– In the case where a shook swarm is applied, TSS should be preferred to PSS to prevent the persistence of *M. plutonius* in the storage combs. Before the shook swarm, healthy colonies must be separated from the infected ones to reduce the risk of cross-contamination. The colonies should not be fed after the shook swarm, in accordance with the principles of cura famis.

## 5. Conclusions

The IMP applied during the experiment to control EFB resulted in a low survival rate in the colonies. Instead of queen caging for a few days after the PSS, we suggest making a very short, temporary queen segregation in proper containers (e.g., queen cages) for a few minutes, just for the time period that is needed to apply the PSS [45]. 

Neither of our IPM strategies applied to control EFB represented an optimal solution. 

If QC is adopted during the swarming season (spring), several side effects adversely impacting the colonies might occur.

However, it must be emphasized that the adverse climatic conditions experienced during the field experiment might have impacted the results of the IPM strategies negatively, being responsible either partially or fully for the outcome of the interventions applied. 

PSS, when applied along with the temporary segregation of the queen for a few minutes, and the separation of treated sick colonies from the untreated healthy ones, might still represent an effective management technique for reducing the amount of antibiotic residues in the honey, allowing it to drop below the MRL within the same productive period. Further studies are warranted to verify the efficacy of TSS carried out without applying QC.

## Figures and Tables

**Table 1:** Colony strength based on the number of frames covered by adult honey bees and by brood before the treatment (Pre) as well as one week (1w), one month (1mo), and 2 months (2mo) after the last treatment, respectively.

	Number of Frames Covered by Adult Honey Bees		Number of Frames Covered by Brood	
	Untreated (Control) Group	OTC-Treated Group	Untreated (Control) Group	OTC-Treated Group
Pre	8.4 ± 1.8	8.7 ± 1.5	5.8 ± 1.2	6.2 ± 1.3
1w	4.1 ± 2.1	3.8 ± 1.9	1.3 ± 1.0	1.0 ± 1.0
1mo	5.0 ± 2.8	5.5 ± 1.8	4.2 ± 2.7	3.8 ± 1.6
2mo	8.6 ± 1.3	6.1 ± 2.1	6.2 ± 0.8	4.8 ± 1.4

**Table 2:** Changes in colony strength based on the number of frames covered by adult honey bees (“Bees”) as well as by brood (“Brood”) based on the results of each sampling time: before the treatment (Pre) as well as one week (1w), one month (1mo), and 2 months (2mo) after the last treatment, respectively. The *p*-values are given both for the untreated group (not marked) and for the OTC-treated groups (marked with ‡) in case there is a significant difference between the colony strength measured at two different time points during the monitoring period.

	Pre		1w		1mo		2mo	
	Bees	Brood	Bees	Brood	Bees	Brood	Bees	Brood
Pre	–	–	*p* < 0.003	*p* < 0.002	*p* < 0.024	Not significant	*p* < 0.011	*p* < 0.026
	–	–	*p* < 0.003 ‡	*p* < 0.003 ‡	*p* < 0.007 ‡	*p* < 0.007 ‡	*p* < 0.011 ‡	*p* < 0.026 ‡
1w	*p* < 0.003	*p* < 0.002	–	–	*p* < 0.011	*p* < 0.007	Not significant	–
	*p*< 0.003 ‡	*p* < 0.003 ‡	–	–	*p* < 0.034 ‡	*p* < 0.007 ‡	Not significant ‡	–
1mo	*p* < 0.024	Not significant	*p* < 0.011	*p* < 0.007	–	–	–	Not significant
	*p* < 0.007 ‡	*p* < 0.007 ‡	*p* < 0.034 ‡	*p* < 0.007 ‡	–	–	–	Not significant ‡
2mo	*p* < 0.011	*p* < 0.026	–	–	Not significant	Not significant	–	–
	*p* < 0.011 ‡	*p* < 0.026 ‡	–	–	Not significant ‡	Not significant ‡	–	–

**Table 3:** Side effects of the PSS combined with the QC on the colony observed in both groups.

Side Effects of the Queen Caging	Group	Colony	Total
Queen loss	OTC-treated group	6/13 (46.2%)	13/25 (52%)
	Untreated group	7/12 (58.3%)	
Absconding	OTC-treated group	2/13 (15.4%)	2/25 (8%)
	Untreated group	0/12 (0%)	
Drone mother colony	OTC-treated group	0/13 (0%)	1/25 (4%)
	Untreated group	1/12 (8.3%)	

**Table 4:** Clinical presence of *M. plutonius* in the OTC-treated and untreated colonies (number of positive cases/number of hives in the respective group; also given as percentages).

EFB Clinical Relapses	4 Months	7 Months
OTC-treated group	0/13 (0%)	0/13 (0%)
Untreated Group	2/12 (17%)	0/12 (0%)

## Data Availability

Data contained within the article.
